# Individual-specific networks for prediction modelling – A scoping review of methods

**DOI:** 10.1186/s12874-022-01544-6

**Published:** 2022-03-06

**Authors:** Mariella Gregorich, Federico Melograna, Martina Sunqvist, Stefan Michiels, Kristel Van Steen, Georg Heinze

**Affiliations:** 1 Section for Clinical Biometrics, Center for Medical Statistics, Informatics and Intelligent Systems, Medical University of Vienna, Vienna, Austria; 2 Division of Nephrology and Dialysis, Department of Internal Medicine III, Medical University of Vienna, Vienna, Austria; 3 BIO3 Laboratory for Systems Medicine, Department of Human Genetics, KU Leuven, Leuven, Belgium; 4 Service de Biostatistique et d’Epidémiologie, Gustave Roussy, Oncostat U1018, Inserm, University Paris-Saclay, labeled Ligue Contre le Cancer, Villejuif, France; 5 BIO3 Laboratory for Systems Genetics, GIGA-R Medical Genomics, University of Liège, Liège, Belgium

**Keywords:** Individual-specific network, Prediction, Personalized medicine, Graph theory, Methodological review, Network analysis, Genomics, Neurology, Pathopsychology, Biomarker

## Abstract

**Background:**

Recent advances in biotechnology enable the acquisition of high-dimensional data on individuals, posing challenges for prediction models which traditionally use covariates such as clinical patient characteristics. Alternative forms of covariate representations for the features derived from these modern data modalities should be considered that can utilize their intrinsic interconnection. The connectivity information between these features can be represented as an individual-specific network defined by a set of nodes and edges, the strength of which can vary from individual to individual. Global or local graph-theoretical features describing the network may constitute potential prognostic biomarkers instead of or in addition to traditional covariates and may replace the often unsuccessful search for individual biomarkers in a high-dimensional predictor space.

**Methods:**

We conducted a scoping review to identify, collate and critically appraise the state-of-art in the use of individual-specific networks for prediction modelling in medicine and applied health research, published during 2000–2020 in the electronic databases PubMed, Scopus and Embase.

**Results:**

Our scoping review revealed the main application areas namely neurology and pathopsychology, followed by cancer research, cardiology and pathology (*N* = 148). Network construction was mainly based on Pearson correlation coefficients of repeated measurements, but also alternative approaches (e.g. partial correlation, visibility graphs) were found. For covariates measured only once per individual, network construction was mostly based on quantifying an individual’s contribution to the overall group-level structure. Despite the multitude of identified methodological approaches for individual-specific network inference, the number of studies that were intended to enable the prediction of clinical outcomes for future individuals was quite limited, and most of the models served as proof of concept that network characteristics can in principle be useful for prediction.

**Conclusion:**

The current body of research clearly demonstrates the value of individual-specific network analysis for prediction modelling, but it has not yet been considered as a general tool outside the current areas of application. More methodological research is still needed on well-founded strategies for network inference, especially on adequate network sparsification and outcome-guided graph-theoretical feature extraction and selection, and on how networks can be exploited efficiently for prediction modelling.

**Supplementary Information:**

The online version contains supplementary material available at 10.1186/s12874-022-01544-6.

## Introduction

Prediction modelling is essential to an individualized approach to risk assessment, diagnosis, prognosis, and medical decision-making. While the conventional approach of model development is mainly based on a small set of patient characteristics, recent developments in biotechnology (e.g. high-resolution imaging modalities and high-throughput sequencing methods) have accelerated the generation of individual-specific data at an unprecedented level generally characterized by a high-dimensional variable space for each individual and complex correlation structures between the variables. Common methods of prediction modelling are not well suited to deal with such complex data structures in particular in small to moderately sized studies [1–3]. Hence the question arises how the abundance of biological information available per individual can be used most efficiently to provide accurate predictions of health outcomes.

**Fig. 1:**
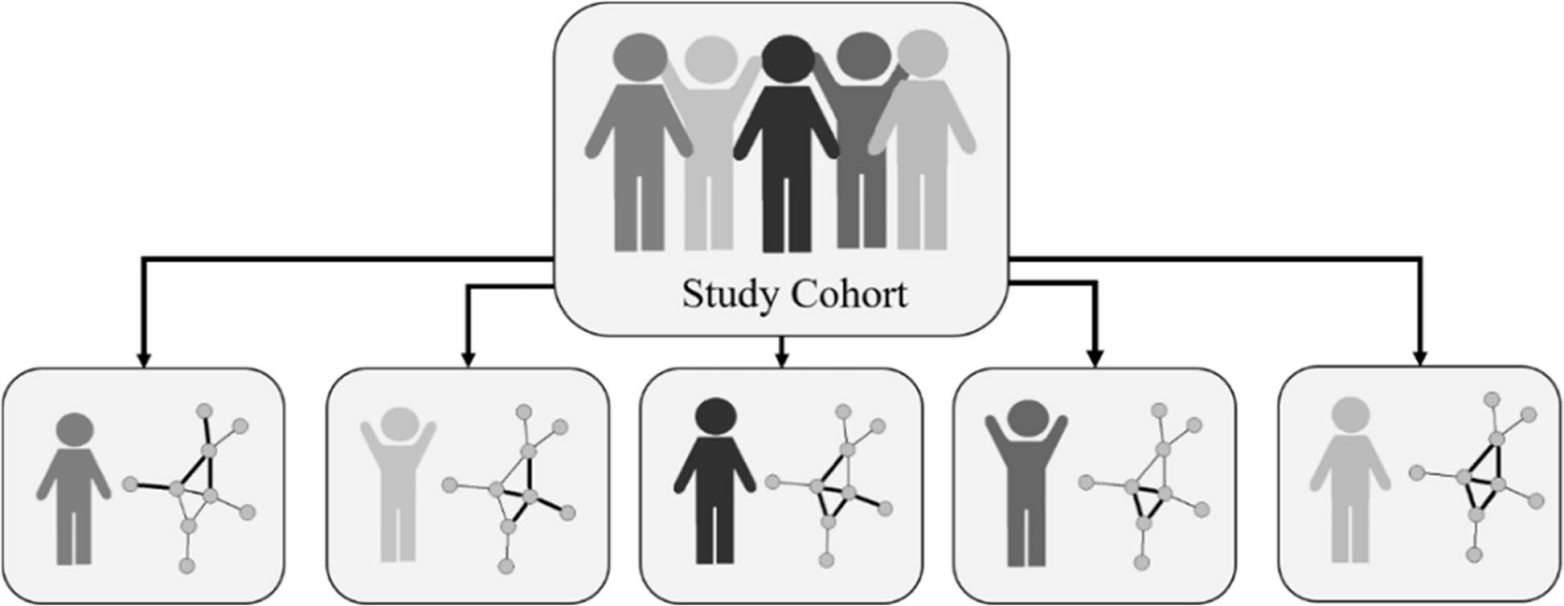
Illustration of the potential heterogeneity inherent in 5 individual-specific networks derived from a study cohort of sample size 5. Line thickness between a pair of nodes indicates the strength of the association (edge weight) within that specific network due to individual-specific variable measurements

Increasingly, it becomes possible to represent individual patient data as individual-specific networks that, apart from individual-specific node measurements, allow to capture connectivity information between variables (nodes) via their edges. Individual-specific networks are exemplified in Fig. 1 for a hypothetical small study cohort. The absence/presence of an edge, or its weight (strength of the connectivity) can be the same for all individuals in the sample (e.g. based on a reference or on sample estimates of a statistical measure of connectivity), or can vary from individual to individual. For example, the individuals depicted in Fig. 1 share the same network structure but are heterogeneous with respect to the edge weights. The network representation is further motivated by subject matter, since complex diseases of the human body are rarely caused by the malfunction of individual molecules but rather by the disruption or dysfunction of the underlying system behaviour or a specific set of biological units [4]. Graph-theoretical features can then capture the heterogeneous variability of system patterns across individuals by describing individual-specific structural and topological network patterns or identify biological modules, a set of nodes acting as key drivers of disease manifestation. For the sake of clarity, variables that originate from individual-specific networks will be referred to as graph-theoretical features throughout this work in order to distinguish them from classic clinical variables. Graph-theoretical features condense a high-dimensional predictor space into few quantitative and interpretable descriptors that can be used in prediction models instead of or in addition to classical clinical predictors in order to improve such models. Such an approach may lead to new insights into disease development or progression and may even replace the often unsuccessful search for individual prognostic biomarkers in the high-dimensional space.

In the past decades, the call for a complex system-based understanding of human disease mechanisms has led to a general theory and various application frameworks of group-level networks, i.e. network inference based on the aggregated study cohort. For example, see Barabási et al. [4] for an extensive review on ‘network medicine’ and Li et al. [5] for an overview of graph representation learning in biology and medicine. However, a “one size fits all” approach for network inference may wash out the individual-specific systems behaviour. Linking individual-specific patterns of connections rather than group-level system behaviour across biological components to disease manifestation can not only capture the heterogeneity of biological system behaviour across individuals but also pave the way for the detection of novel biomarkers for more individualized prediction.

To our knowledge, the potential of using graph-theoretical features of individual-specific networks as predictors in clinical prediction models has not yet been systematically explored and hence the state-of-the-art in this relatively new field of predictive research remains unknown. Therefore, we conducted a scoping review [6, 7] to systematically examine the scientific literature to identify, collate and critically appraise methodological approaches incorporating individual-specific networks to improve and advance prediction modelling of clinical outcomes in applied health research. We did not consider studies exclusively employing group-level networks and those which do not aim at individual outcome prediction.

The remainder of this paper is organized as follows. In Section 2, we discuss the methodology of the scoping review. The general study characteristics and findings of the search strategy are presented in Section 3. Further, we present and collate the identified approaches to individual-specific network inference, graph-theoretical feature extraction and their usage for prediction in Section 4 and conclude with current challenges and future aspects of the identified methodology in Section 5.

## Methods

### Search strategy

We conducted a scoping literature search in the three electronic databases PubMed, Embase and Scopus to extract peer-reviewed articles published between January 1st, 2000 and August 31st, 2020. The search strategy consisted of three sets of terms to cover the research intersection of network analysis and prediction modelling adequately but also to reduce false positive hits by the broad meaning of the term ‘network’. The three sets of terms were: 1) terms associated with network analysis, 2) terms associated with predictive research and 3) exclusion terms (see the Supplementary Material for a detailed overview).

Studies met the inclusion criteria if graph-theoretical features derived from an individual-specific network were considered as candidate predictors in prediction modelling in the medical field. Studies were excluded if they did not focus on prediction modelling of health outcomes and did not consider networks constructed for single individuals. Therefore, we excluded studies that concentrated solely on the descriptive analysis of individual-specific networks without examining their potential association with a clinical outcome or studies considering group-level networks computed from aggregated data. Studies that essentially aimed at predicting network behaviour, link prediction or changes in network topology or structure were also discarded.

### Selection of studies

All studies identified by the search strategy were initially screened based on the title and then, after inclusion, based on the abstract to determine eligibility. Letters, commentaries and conference abstracts were excluded. Selected articles were then subjected to a full-text analysis. The first author was responsible for the initial search, application of the exclusion criteria, screening of all the identified articles and the quality evaluation of the included papers. A random subset of studies (consisting of 250, 50 and 25 studies in the title, abstract and full-text screening phases, respectively) was independently assessed by three additional reviewers (GH, FM, MS) to ensure general validity and reliability of the screening process and data extraction of the first reviewer. Any inconsistencies in selection among the reviewers were discussed and resolved to reach a general consensus., We refer the reader to the Supplementary Material for a more detailed summary of the search strategy and the extraction process. Reporting adhered to the PRISMA-ScR guidelines [7] to ensure methodological transparency.

## Results

### Search results and study characteristics

**Fig. 2:**
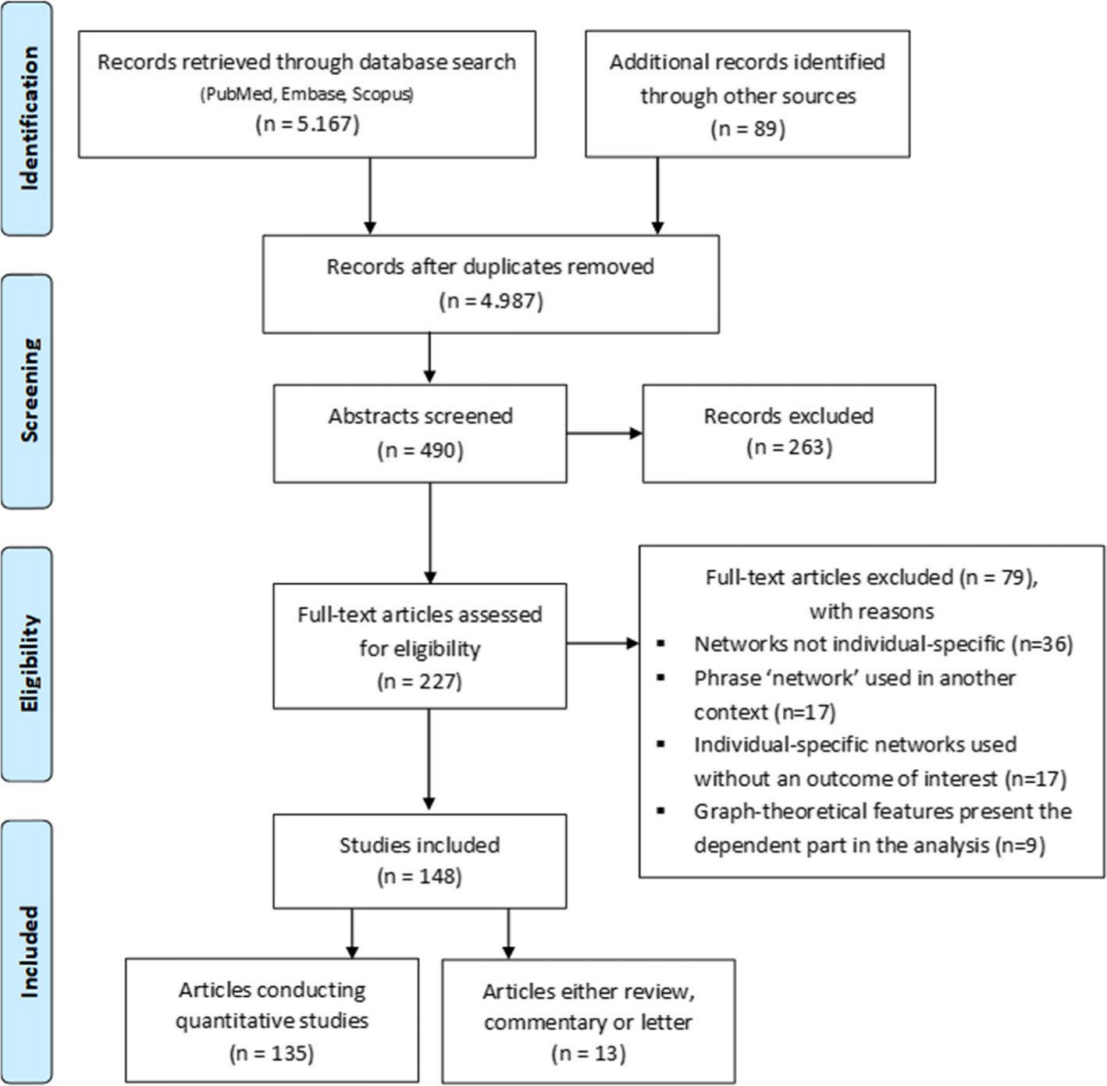
PRISMA flowchart for the scoping review of articles retrieved by the search strategy

A total of 4988 studies was initially retrieved from the electronic database search together with the manual selection from other sources. After the screening of the titles, 488 articles remained and after reviewing the abstracts, only 227 articles met the eligibility criteria for full-text analysis of which 79 were excluded due to the following reasons: (1) construction of non-individual-specific networks (*N* = 36), (2) the term “network” was used in a different context (*N* = 17), (3) no association with an outcome of interest was considered (*N* = 17) or (4) graph-theoretical features were used as dependent variables (*N* = 9). This left 148 studies (3.0%) out of the initial 4988 studies meeting the eligibility criteria of the review (see Fig. 2).

**Fig. 3:**
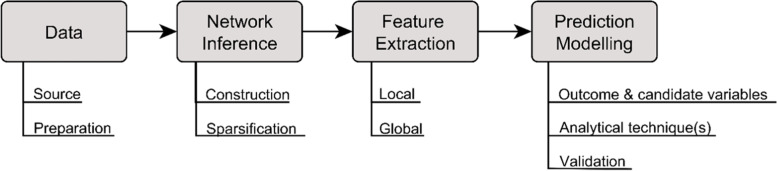
Main modules of a workflow considering individual-specific networks in prediction modelling and their related aspects of implementation

Through a synthesis of the sources of evidence, four medical domains of application were identified. The majority of the eligible studies (*N* = 129, 87.2%) covered neurological research, followed by the fields of psychopathology (*N* = 9, 6.1%), genomics (*N* = 7, 4.7%), cardiology (*N* = 2, 1.4%) and pathology (*N* = 1, 0.7%). The oldest study meeting our inclusion criteria was published in 2009, with the number of studies published annually increasing steadily thereafter (see Supplementary Fig. S1). Most of the studies (*N* = 100, 67.6%) were published after 2015. Out of the 148 included studies, 135 (91.2%) were identified as quantitative studies and 13 (8.8%) as qualitative studies (e.g. reviews). Besides the process of data acquisition and preparation in applied health research, three main topics emerged in the included articles covering the intersection of network analysis and prediction and were addressed separately in the following subsections: Section 3.2 focuses on data-driven network inference consisting of the individual-specific network construction and network sparsification, Section 3.3 on the extraction of graph-theoretical features and Section 3.4 on predictive analytics using graph-theoretical features. Main modules in a general workflow of considering individual-specific networks in prediction modelling and some aspects of their implementation are illustrated in Fig. 3. However, a detailed presentation of each of the identified analytics for network analysis and prediction modelling as well as their theoretical properties is beyond the scope of this article. Instead, the reader will find useful references throughout this work.

### Network construction and sparsification

#### Notation and concepts

In general, an undirected network (or graph) consists of a pair *G* = (*V*, *E*) where *V* denotes a finite, non-empty set of *p* nodes and *E* is a subset of *V* × *V* containing pairs of connected nodes *e*_*ij*_ ≔ (*v*_*i*_, *v*_*j*_) referred to as edges. In directed graphs (digraph), each edge has a direction such that *e*_*ij*_ ≠ *e*_*ji*_. In weighted networks, each edge *e*_*ij*_ is associated with a weight *w*_*ij*_ ≔ *w*(*v*_*i*_, *v*_*j*_) ∈ *ℝ*. A subnetwork *G*^′^ = (*V*^′^, *E*^′^) is a network such that *V*^′^ ⊆ *V* and *E* ′  ⊆ *E*. The data structure defining a network is the adjacency matrix *A* = [*a*_*ij*_] in which *a*_*ij*_ = 1 indicates the presence of an edge between *v*_*i*_ and *v*_*j*_, while *a*_*ij*_ = 0 indicates its absence. For weighted networks *a*_*ij*_ = *w*_*ij*_, and again *a*_*ij*_ = 0 indicates the absence of the respective edge. For individual-specific networks, we assume that for each individual *s* (*s* = 1, …, *N*) a unique network *G*_*s*_ = (*V*_*s*_, *E*_*s*_) exists, where *N* is the number of individuals within the study cohort .

Global graph-theoretical features characterize properties of the entire network, while local features only take the information of a smaller substructure of the network (e.g. node, module) into account. For example, edge density is a global graph-theoretical feature defined as the ratio of the number of actual connections to the number of all possible connections in the network

1 $$d=\frac{2\mid E\mid }{\mid V\mid \left(\left|V\right|-1\right)}$$

See Table 1 for an overview of some global and local graph-theoretical features identified in the reviewed studies.

**Table 1:** Explanation of the graph-theoretical features used in ≥10 prediction modelling studies. Local features can be averaged over all nodes to obtain the global-scale counterpart

Graph-theoretical feature	Abbrev.	N	Scale	Explanation
Clustering coefficient	CC	82	Both	Ratio of the connected triangles to the maximum possible number of triangles
Characteristic path length	CPL	60	Both	Average of all shortest paths over all pairs of nodes
Global efficiency	GE	55	Global	Average of the reciprocals of the shortest path lengths
Local efficiency	LE	45	Local	Global efficiency applied to the neighbourhood of a node
Small-world index	SWI	42	Global	Ratio of the CC normalized by that expected in a random graph and the CPL normalized by that expected in a random graph
Degree	Dg	38	Local	Number of links of a node
Betweenness centrality	BC	36	Local	Ratio of all shortest paths with and without the node
Edge weight	EW	34	Local	Strength of the connection between two nodes
Modularity	M	21	Global	Degree to which nodes tend to form relatively independent modules
Density	Ds	18	Global	Percentage of observed connections from the maximum number of possible connections
Assortativity	A	10	Global	Pearson correlation coefficient of degree between pairs of connected nodes

#### Construction of individual-specific networks

##### Repeated measurements per variable per individual

**Fig. 4:**
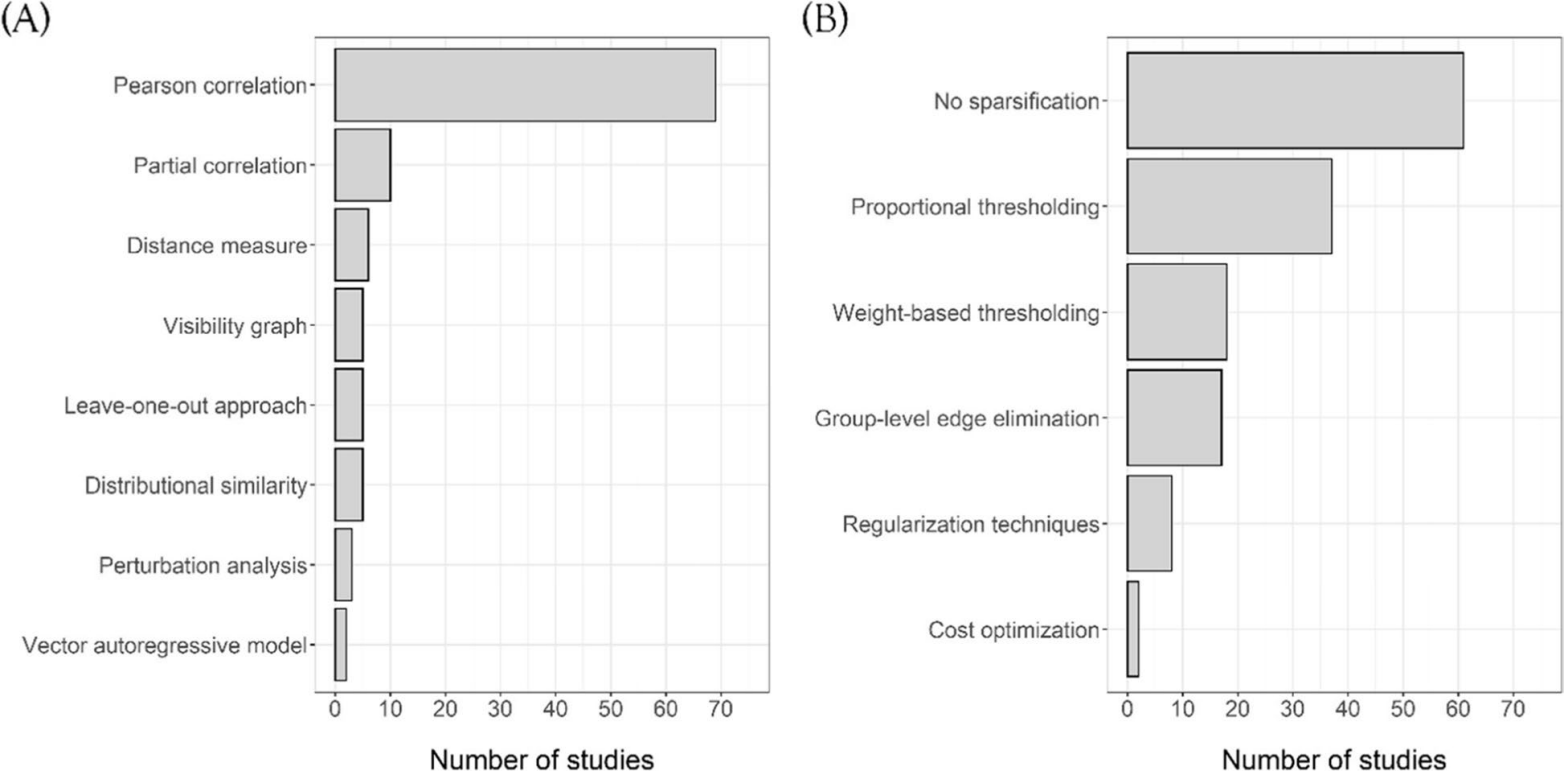
Distribution of (**A**) individual-specific network construction and (**B**) sparsification approaches in the identified quantitative studies

Depending on the field of research, the nodes mainly represented regions of interest (ROIs) in the brain, genes or psychotic symptoms (e.g. stress, insomnia). In two individual proof-of-concept studies, the nodes corresponded to 5-min heart rate variability (HRV) segments [8] or ROI in muscle biopsy images [9]. A variety of methods were identified for defining connectivity between pairs of nodes based on data-driven structural learning, i.e., estimating the graphical structure from the data (see Fig. 4 for a schematic illustration).

The majority of identified studies conducted correlation-based approaches (see Fig. 4A) using repeatedly measured continuous data (e.g. sequential data, time series) to define edges between the prespecified nodes [10–27]. In neurological applications, adjacency matrices most frequently consisted of correlation coefficients (*N* = 69, *P* = 48.9%) (e.g., Pearson product moment correlation coefficient) of time series of brain activity (e.g. blood oxygenation level dependent (BOLD) signal) or longitudinal cortical thickness between pairs of ROIs [16]. Fisher’s r-to-z transformation was applied to Pearson correlation coefficients to achieve approximate normality [14, 21, 26, 28, 29]. However, correlation-based inference of the network structure can be subject to interfering effects (e.g. outliers, noise) and can only capture pairwise information. Thus, variations of the Pearson correlation such as spatial smoothing have been proposed in which the time series corresponding to a region is obtained by a linear mixture of neighbouring time series or segmentation of the BOLD time series into subdivisions (e.g. snapshot graphs, sliding-window approach) [10, 30–32]. The sliding window or snapshot graph approach only consider small time windows of the full time series yielding a set of graphs *G*_*s*_ = {*G*_*s*1_, …, *G*_*st*_} for individual *s* and *t* time windows. Then, a presumably more robust final version of the individual-specific network can be obtained by assessing the frequency of appearance of each edge in each *G*_*sj*_ for *j* = 1, . . , *t* because only a few of these snapshot graphs *G*_*sj*_ are influenced by disruptions of the time series by noise artefacts [10]. Some studies also employed several modifications of the standard Pearson correlation coefficient to define connectivity [33–37].

An extension to the bivariate determination of connectivity were partial correlation-based networks [38, 39]. Partial correlation coefficients describe the correlation between two variables that cannot be explained by associations of the variable pair with other variables. In the case of non-normality of the data, a transformation can be applied prior. Another approach to control for covariates while accounting for temporal information is vector autoregressive (VAR) modelling that regresses the dependent variable measured at time point *t* on the lagged dependent variable and the predictors evaluated at time point *t* − 1. VAR was only employed in psychological studies [40, 41]. This way directed individual-specific networks were obtained such that an edge weight *w*_*ij*_ associated with a directed link from node *v*_*i*_ to node *v*_*j*_ corresponds to the respective VAR coefficient in the model. The VAR model and resulting directed networks implied that in general *w*_*ij*_ ≠ *w*_*ji*_. Only 2 out of the 135 quantitative studies considered directed networks estimated using VAR models [40, 41].

A recently introduced network-based representation referred to as a visibility graph allows to derive individual-specific networks from time series data if available per patient in the cohort [42]. A time series with *n* sequentially ordered data points, {*x*_*t*_}_*t* = 1, …, *n*_ is transformed into a network in which each time point *t* represents a node connected to time point *s* if the visibility criterion

$${x}_u<{x}_s+\left({x}_t-{x}_s\right)\frac{s-u}{s-t}$$

holds true for an additional data point *x*_*u*_ placed between them. Applications of visibility graphs were mostly found in context of EEG data [43–45] but also in a cardiologic study dealing with human heart rate variability time series to differentiate between wake/sleep stages [46] and patients of spinal cord injury [47]. A more thorough overview of concepts and algorithms to map time series data into networks can be found in Silva et al. [48].

Alternatively, various distributional similarity approaches were used to evaluate the similarity between two distributions corresponding to a pair of nodes (Jensen-Shannon divergence [49], Kullback-Leibler divergence [50], dynamic time warping [8], generalized measure of association [51]). Zhang et al. [49] employed a kernel-based on the Jensen-Shannon divergence to measure the similarity of multivariate time series, whereas Dong et al. [8] assessed similarity between pairs of time series based on dynamic time warping.

##### Single measurement per variable per individual

The approaches presented so far are only applicable if repeated data points of all variables per individual are available. In the identified studies in which all independent variables were measured only once for an individual, individual-specific network inference was mainly based on quantifying each individual’s contribution to the overall group-level structure either by leave-one-out network construction (LOONC) [52, 53] or by a differential perturbation approach [54–59]. Strictly speaking, these two approaches do not involve individual-level parameter inference per se but aim to derive individual-specific networks from group-level networks regardless of the group-level network inference procedure. More precisely, LOONC removes a single individual from group-level network construction and measures the degree of change in all edge weights caused by the removal against the full group-level network. Kuijjer et al. [52] proposed to reversely engineer individual-specific networks by linear interpolation between each edge weight ${w}_{ij}^{\left({C}_{int}\right)}$ of the group-level network (using all ${N}_{C_{int}}$ observations in the study cohort of interest *C*_*int*_) and the corresponding edge weight ${w}_{ij}^{\left({C}_{int}\backslash q\right)}$ in the network using all observations except an individual of interest *q* to obtain the edge weight estimate ${w}_{ij}^{(q)}$ for a single individual by

2 $${w}_{ij}^{(q)}=N\left({w}_{ij}^{\left({C}_{int}\right)}-{w}_{ij}^{\left({C}_{int}\backslash q\right)}\right)+{w}_{ij}^{\left({C}_{int}\backslash q\right)}$$

where *N* can be a generic or individual-specific weight but is usually set to ${N}_{C_{int}}$. In contrast, differential perturbation analysis quantifies the extent to which an edge weight is perturbed by the addition of an individual compared to a reference network constructed independently from the cohort of interest. Edge weights of the individual-specific network *G*_*q*_ for individual *q* are determined by computing the absolute difference in edge weights between the network computed from the reference population augmented by that individual (augmented network) and the reference network as

3 $${w}_{ij}^{(q)}={w}_{ij}^{\left({C}_{ref}\cup \mathrm{q}\right)}-{w}_{ij}^{\left({C}_{ref}\right)}$$

where ${w}_{ij}^{C_{ref}}$ denotes the edge weights in the reference network and ${w}_{ij}^{\left({C}_{ref}\cup \mathrm{q}\right)}$ in the augmented network.

Neither of the two presented approaches to individual-specific network construction for single measurements of each variable per individual relies on a particular inference method for the group-level networks. Equation (2) and (3) require the edge weights of the estimated group-level networks but do not specify how these networks have to be inferred. Perturbation analysis in contrast to LOONC, however, depends on an additional reference network to obtain individual-specific edge weights.

In the study by Zhu et al. [54], a group-level reference network for the differential perturbation analysis was inferred using Pearson correlation coefficients of gene co-expression data of ${N}_{C_{ref}}$ cancer-free individuals of a reference cohort *C*_*ref*_. An augmented network for an individual with cancer *q* was constructed by including the expression data of that individual in the reference cohort (*C*_*ref*_ ∪ *q*). The approach was also found with networks constructed using partial correlation [57]. In Kuijjer et al. [52], LOONC was implemented using group-level networks derived from Pearson correlation and mutual information, a non-linear measure of association, while Lopes-Ramos et al. [53] used biologically motivated regulatory networks. Note that since both equations quantify the inferred difference in edge weighting, the obtained individual-specific weights can also assume values outside the boundaries of the underlying statistic of the group-level networks. For example, edge weights in the individual-specific networks derived from LOONC or differential perturbation using Pearson correlation can yield values <− 1 and > 1 (see Eq. (2–3)). Both approaches were only found in cancer research [57, 59].

Only one of the identified studies using single measurements per variable did not conduct LOONC or perturbation analysis. Xie et al. [39] proposed conditional Gaussian graphical modelling with mean and covariance matrix depending on an individual’s covariates while assuming homogeneous network structure across individuals in order to infer individual-specific networks. Hence, differences in edge weights between individuals are covariate-dependent.

##### Domain-specific techniques

In neurological application, certain high-resolution imaging methods for data acquisition generate domain-specific data structures that require a specifically designed methodology of network inference. For instance, tractography detects fibre pathways linking different anatomical brain regions [60–68]. Electroencephalographic (EEG)-based connectivity was predominantly assessed by metrics such as phase lag index, coherence-based similarity, or synchronization likelihood index [69–72]. The interested reader is referred to the references for more details on these applications.

#### Techniques for network sparsification

Network sparsification removes edges from a network with the intent to optimise the inference of the ‘true’ system by the omission of “spurious” edges, improve interpretability and enable computational feasibility in construction and further processing of the network. In addition, the structural and topological properties of the network might be improved by removing seemingly spurious connections i.e. edges that are erroneously generated during network inference. Given an undirected network *G* = (*V*, *E*), sparsification yields a subnetwork *G* ’  = (*V*’, *E*’) of *G* with fewer edges such that |*E*| >  ∣ *E*^′^∣.

**Fig. 5:**
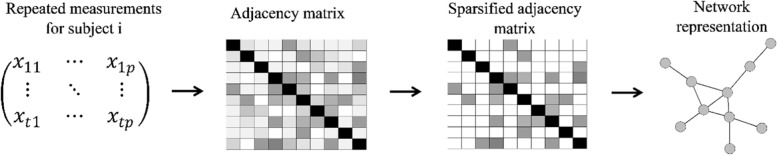
Schematic overview of network inference with network construction-based on repeated measurements of p variables of an individual i in the study cohort and sparsification using a single threshold value

More than half of the quantitative studies employed network sparsification (*N* = 74, 54.8%), followed by studies analysing non-sparsified networks (*N* = 58, 43.0%) and 3 studies that considered both approaches (2.2%). As illustrated in Fig. 4B, the most popular strategy was proportional thresholding (*N* = 37) in which a common sparsity threshold (e.g. in terms of density) is defined for all individual-specific networks, and edges are removed sequentially in each network in ascending order of their edge weights until the prespecified sparsity threshold is reached [10, 21, 23–25, 29, 58, 73]. The second most common approach constituted weight-based thresholding (*N* = 31) in which one common weight threshold is defined for all individual-specific networks such that all edge weights within an individual-specific network falling below (or above) the threshold are removed (Fig. 5) [8, 11, 67, 68, 74, 75]. The majority of the weight-based approaches employed binarization of all individual-specific networks (*N* = 21) according to the selected weight threshold *τ* such that ${w}_{ij}^{\prime }=1$ if *w*_*ij*_ > *τ* and 0 otherwise [23, 27, 29, 75, 76]. Edges with negative weights were often removed due to their questionable interpretability or the inability to compute some graph-theoretical features from them (e.g. clustering coefficient or characteristic path length), omitting potentially meaningful inverse relation between nodes. In *N* = 6 studies, a sparse representation of a partial correlation-based network was achieved individually by including an *L*_*q*_-norm (*q* ∈ {1, 2}) regularization penalty to the network inference process in order to reinforce strong connections across individuals and drive weak connections towards zero [36, 37, 39, 77, 78]. Due to the individually imposed regularization, inter-subject variability might then be inherently induced according to Wee et al. [36]. Hence, they imposed an additional group constraint to encourage a common network topology across the study cohort. Other studies selected a fixed penalty term for all individual networks which is why this approach can also be seen as a special case of weight-based thresholding [34, 37]. Group-level edge elimination for each individual-specific network was carried out by univariate testing of the edge weights (*N* = 17), either by permutation testing to control the probability of including spurious connections at 0.05 or by only including edges with weights significantly different from zero [13, 15–19, 79–81]. Then all those edges that were characterized as spurious at the group level were removed from all individual-specific networks.

**Fig. 6:**
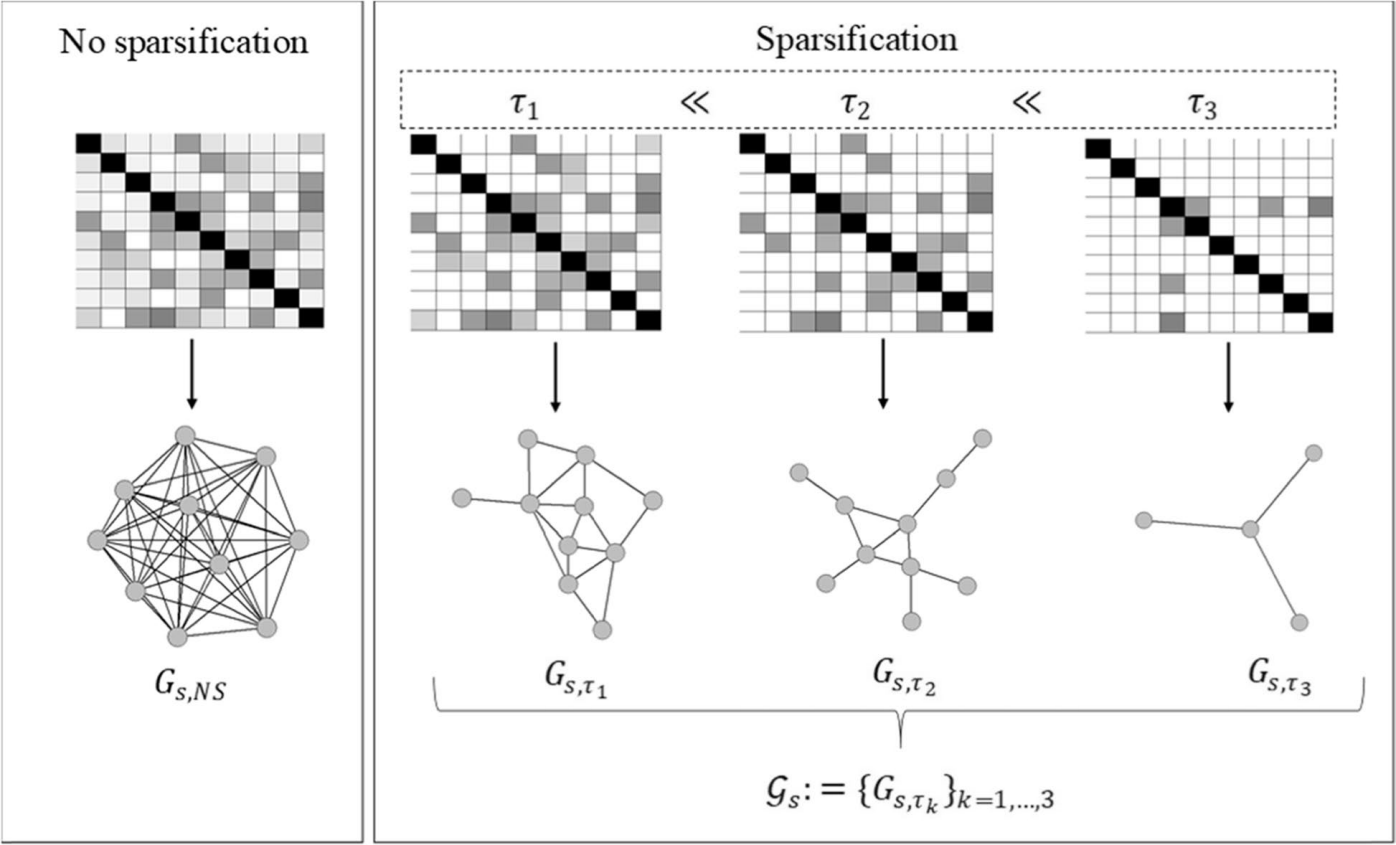
The variability of an individual-specific network for an individual s depending on three different threshold values with τ_1_ ≪ τ_2_ ≪ τ_3_ yielding the network set ${\mathcal{G}}_{\mathrm{s}}$ in comparison with no sparsification

The implemented strategy of network sparsification across studies varied substantially in terms of: the sparsification method, the number or combination of investigated methods, the selected sparsification threshold(s) and the reasoning behind the chosen strategy. It was not uncommon for studies to combine several sparsification strategies [12, 21, 22, 24] or to examine several strategies separately [33, 34, 37]. Further, all of the identified approaches to network sparsification were dependent on the careful selection of a thresholding parameter by the researcher. To circumvent the arbitrariness associated with the selection of a single threshold, the majority of studies employed multiple thresholding, meaning that a range of cut-off values with small incremental steps was examined such that a series of networks ${\mathcal{G}}_s:= {\left\{{G}_{s,{\tau}_k}\right\}}_{k=1,\dots, \mathrm{T}}$ for each individual *s* (*s* = 1, …, *N*) was obtained with *T* thresholds *τ*_*k*_ where *k* = 1, …, *T* [65, 73, 76, 82]. For an illustrated example of multiple thresholding, see Fig. 6. Only one study performed weight-based thresholding with a single, arbitrary threshold [83].

In general, there was considerable heterogeneity in the selection of the optimal threshold value for sparsification in the studies evaluated:(1) a threshold yielding a small-world index (see Table 1 for definition) above 1 of the networks was chosen [65, 84], (2) an arbitrary fixed threshold was chosen [39], (3) the differently sparsified networks corresponding to a single individual ${\mathcal{G}}_s$ were fused into an average individual-specific network [25], (4) the numerical integral or average of the graph-theoretical feature over the range of network ${\mathcal{G}}_s$ was computed [25, 56, 64, 65, 76], (5) a threshold generating the best results in terms of classification accuracy or association with the outcome was selected [8, 29, 34, 51], and (6) further processing was done including varying network sparsity levels [24, 75, 85].

According to the identified studies conducting network sparsification, the choice of the threshold is crucial to balance between noise removal (spurious edges) and preservation of ‘true’ edges. For instance, proportional thresholding may leave edges with very low edge weights assumed spurious, whereas weight-based thresholding yields different densities across the individual-specific networks which then may affect other graph-theoretical features and in turn, hinder comparability across networks. While the evaluated studies often adequately addressed the selection of the threshold parameter weight-based and proportional thresholding, ‘hidden’ threshold parameters in regularized network inference (i.e. penalty strength) or univariate testing (i.e. significance level) were rarely further investigated.

### Graph-theoretical feature extraction

Appropriate features describing the network after its construction reduce the dimensionality of the network, capture aspects of the graph structure and may constitute valuable biomarkers for clinical outcomes in applied health research. The connectivity information within a network can be characterized on different scales: globally or locally. While global features capture properties of the whole network, local features describe their characteristics in defined subareas such as nodes, edges or modules (i.e. clusters of nodes).

The majority of quantitative studies extracted both, local and global graph-theoretical features (*N* = 56, 41.5%), followed by studies only focusing on local features (*N* = 44, 32.6%) and studies interested only in global graph-theoretical features (*N* = 35, 26.0%). The average number of computed global graph-theoretical features describing different aspects of the network across all studies was 3.19 with a standard deviation (SD) of 4.69 and a range of 0 to 44, while the average number of local features was comparatively small with a mean of 1.62 and a SD of 1.72 and a range of 0 to 10. The most frequently examined graph-theoretical features were the clustering coefficient (CC) (*N* = 84, 62.2%) [86] quantifying the tendency of clustering within the network and the characteristic path length (CPL) (*N* = 61, 45.2%) defined as the *average* of all shortest paths over all pairs of nodes in a network. A total of 57 different graph-theoretical features were identified. The most commonly used metrics (examined in more than 10 studies) are briefly explained in Table 1. More detailed descriptions are available elsewhere [87]. In some studies, graph-theoretical features were normalized by dividing them by the same metric computed from a randomly generated network of identical size, density and/or degree distribution to account for differences in network size and density, introducing additional computational complexity. Largely, studies (*N* = 31, 23.8%) examined the normalized clustering coefficient and characteristic path length obtained by dividing the CC and CPL by the CC and CPL of multiple randomly generated networks [18, 20, 79, 81, 84, 88]. For instance, Imms et al. [89] highlighted the use of normalized CC and CPL as diagnostic biomarkers to differentiate between controls and patients with traumatic brain injury.

### Feature selection and prediction modelling

Around a third of the 135 quantitative studies (*N* = 46, 34.1%) conducted implicit techniques for prediction i.e. elementary statistical analytics (e.g. hypothesis testing, correlation analysis) to identify potential biomarkers associated with the outcome [8, 59, 84, 90] while the remaining studies (*N* = 89, 65.9%) conducted prediction modelling using explicit techniques i.e. methods with the possibility of predicting for an unseen individual. Among the latter, about two thirds aimed at the supervised identification of disease subgroups using graph-theoretical features as independent variables (*N* = 57, 64.0%) and one third prognosticated a clinical outcome (*N* = 32, 36.0%). Despite the similarity of the methodological frameworks, the evaluated studies turned out to be very heterogeneous regarding feature selection, sample size, outcome types, the outcome modelling technique and the validation analytics employed.

#### Feature selection

Feature selection approaches identified in the studies used to define an optimal subset of features can be classified into filter methods (e.g. univariable testing, Pearson correlation) and wrapper methods (iterative optimization of a classification algorithm) but hybrid approaches were also proposed [83]. The majority of studies used filter methods to remove features before training a classifier or a regression model [20, 21, 91]. Wrapper methods (e.g. support vector machine (SVM) with recursive feature elimination (RFE), repeated selection across leave-one-out cross-validation (LOOCV) runs) either iteratively reduced the features based on a ranking score (e.g. feature importance) of the feature in the prediction algorithm to optimize classification accuracy [25, 63] or selected features with the most discriminative ability out of the full set of features based on repeated least absolute shrinkage and selection operator (LASSO) feature selection across LOOCV runs [77].

#### Prediction modelling

**Fig. 7:**
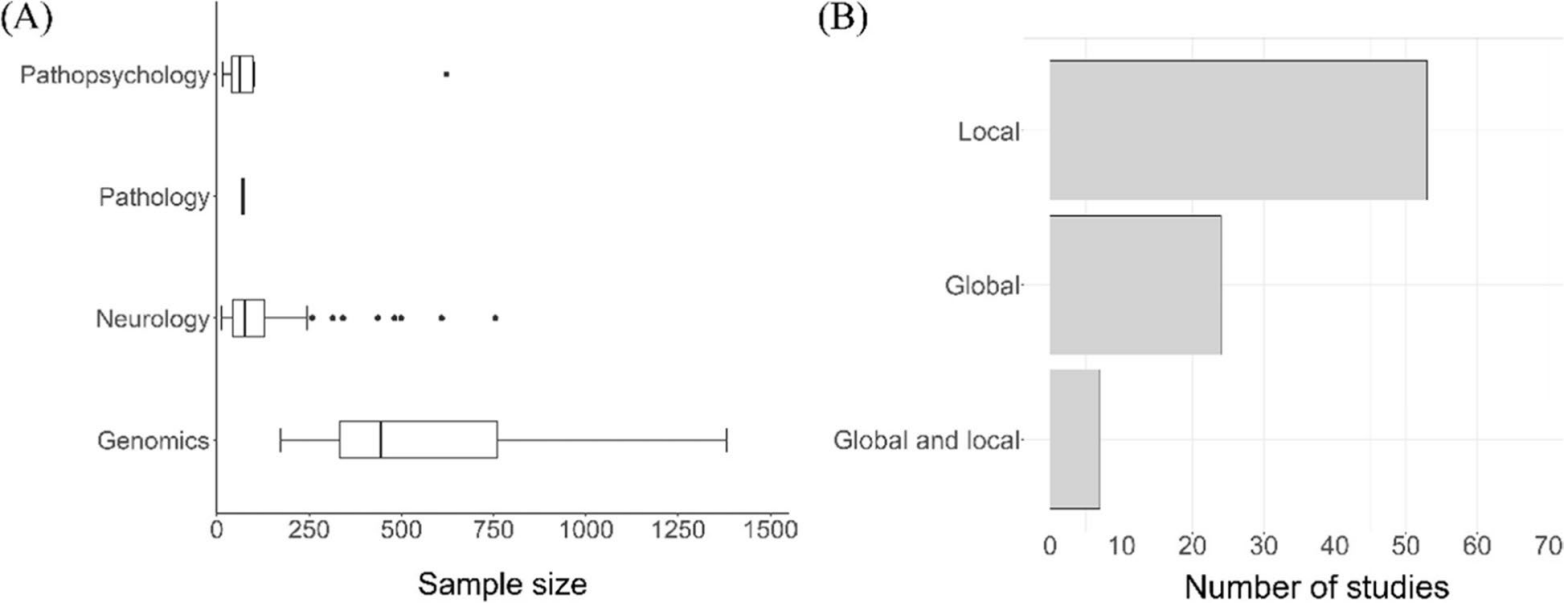
**A** Distribution of sample size across the identified studies; **B** Distribution of type of feature extracted for prediction modelling

The median sample size in neurology was 68 (interquartile range (IQR) 41 to 127), in genomics 445 (IQR: 333–761), and in pathopsychology 62 (IQR: 41–97) as illustrated in Fig. 7A. In pathology, only one study with a sample size of 70 was identified and two studies in cardiology with a sample size of 55 and 389 individuals, respectively. The most commonly investigated types of dependent variables across all 135 quantitative studies were binary (*N* = 86, 63.7%), followed by continuous (*N* = 37, 27.4%), categorical (*N* = 14, 10.4%) and lastly, time-to-event (*N* = 4, 3.0%) with some studies assessing multiple outcome types (*N* = 6, 4.4%). The considered statistical modelling techniques employed for the task of prediction varied accordingly. Hence, for the sake of clarity, we will distinguish between data modelling and algorithmic modelling approaches according to Breiman [92] (Table 2). The former group contains modelling techniques that connect covariates to the outcome variable by a stochastic model (e.g. linear or logistic regression), while approaches belonging to the latter group use an algorithm to predict the outcome from the covariates (e.g. SVM, random forest). For a complete list of the grouping of the identified methods, we refer to the supplementary material. In general, SVMs were the most popular approach (*N* = 40, 45.0%) [27], followed by linear regression (*N* = 25, 28.0%) [19, 22, 28, 64, 67, 81, 93, 94], random forests (*N* = 12, 13.5%) [15, 41, 58, 72, 95, 96] and logistic regression (*N* = 9, 10.1%) [41, 61, 65, 97, 98].

**Table 2:** Types of prediction modelling approach identified in the 89 studies conducting advanced statistical procedures based on graph-theoretical features

	Algorithmic modelling	Data modelling
Total	56	46
Sample size	137.75 (171.46)	168.54 (232.62)
*Graph-theoretical scale*		
Global	9	23
Local	45	20
Both	2	4
*Outcome*		
Continuous	5	21
Binary	46	20
Categorical	5	3
Time-to-event	0	2

*Continuous variables stated as mean (standard deviation, *SD*). Categorical variables represent the number of approaches identified in the studies

The majority of quantitative studies built discriminative classifiers for discrete outcome labels mainly using only local graph-theoretical features (see Table 2 and Fig. 7B). The most common approach extracted local graph-theoretical features with filter-based feature selection and then trained a linear SVM for outcome classification [16, 30, 37, 62, 77, 83]. A popular choice for feature selection before SVM training and classification was univariable feature screening of statistical significance [10, 13, 14, 34, 36, 74, 78]. However, this method is known to suffer from conceptual shortcomings [99]. In the case of multiple thresholding [74], the use of multiple data modalities [14] or the extraction of multiple local features [32, 100] for classification, a supervised multiple-kernel learning approach was adopted to fuse layers of features to predict disease subgroups. A subset of studies even trained multiple classifiers for the binary classification of the presence of disease (e.g. SVM, k-nearest neighbour, decision tree, random forests, Naïve Bayes, Adaptive Boosting) [11, 26, 35, 69, 96, 101]. For instance, [26] stated to have built 67 classifiers to differentiate between children with autism spectrum disorder and age-matched controls and 23 advanced regression models for phenotypic prediction.

In terms of model validation, nearly all diagnostic studies reported model discrimination and performance of the classifier in terms of cross-validated accuracy, sensitivity, specificity and the area under the receiver operating characteristic curve (AUC) [12, 23, 25, 27, 70, 77]. Some studies reported estimated predictive accuracy solely utilizing correlation between the predicted and the true values [93, 96]. In a few studies, performance was claimed to exceed previous approaches, but such a claim was often unsubstantiated and solely based on the comparison between their overall accuracy or AUC and that of the existing studies without consideration of other study factors (study design, set of variables, sample size, heterogeneity between study cohorts) [26, 102, 103]. Imbalanced distribution of class labels was stated as an issue for training accurate classifiers but was accounted for in some studies by assessing the balanced accuracy (i.e. the arithmetic mean of sensitivity and specificity) [58, 70, 74, 100]. Further, calibration analysis as a reliability assessment of the predicted probability of the event actually occurring with the observed relative frequency by the means of a calibration curve was rarely reported in any of the evaluated studies.

Regression-based modelling was mainly performed by linear regression (*N* = 28, 60.7%), followed by logistic regression (*N* = 8, 17.4%), Cox proportional hazards regression (*N* = 2, 4.3%) and mixed-effects modelling (*N* = 2, 4.3%). In case global graph-theoretical features were extracted from each individual-specific network or a continuous outcome type was of interest, regression-based analytics were the preferred course of action (see Table 2). The majority of the studies used only graph-theoretical features as independent variables and rarely adjusted for clinical characteristics (e.g. in [15, 18, 21, 22, 67, 73, 80, 97]). Even fewer studies adjusted for network size and density within the regression model to account for their inter-subject differences affecting the extracted graph-theoretical features [79, 81]. Model adjustment by clinical information was more common in studies investigating the association of global graph-theoretical features with an outcome of interest rather than studies interested in local features. Batalle et al. [61] stated that the graph-theoretical features included in addition to the clinical-epidemiological covariates even proved to yield a higher contribution in terms of statistical significance after separate (blockwise) stepwise backward elimination of variables. However, clinical variables were not only used to adjust the model. For example, Xie et al. [39] proposed a two-stage approach consisting of first, deriving individual-specific network models from conditional Gaussian graphical models dependent on an individual’s clinical features and second, the clinical outcome model to estimate the graph-theoretical parameters’ effects on an outcome of interest adjusted by the same clinical features.

The sample size of studies conducting regression-based methods was slightly higher and showed a significantly greater variance in comparison with the sample size studies employing classification-based methods (Table 2). Furthermore, possible overfitting of predictive data modelling was rarely directly addressed as in the study by Batalle et al. [61] who reduced preselected graph-theoretical features into a single summary index or in Anderson et al. [98] who restricted the variables which entered the model to 4 to have at least 5 events per variables [104].

Only three of the 130 quantitative studies conducted Cox proportional hazards model to model a time-to-event outcome [66, 77, 79]. Tuladhar et al. [66] identified global efficiency instead of conventional MRI biomarkers as a predictor of all-cause dementia with lower global efficiency associated with a higher risk of dementia onset while adjusting for a set of clinical features. Similarly, Liu et al. [77] stated that none of the traditional clinical features was consistently selected in the majority of the LOOCV runs for overall survival time of high-grade glioma patients and out of the three most important selected features, two were graph-theoretical features.

## Discussion

In this scoping review, we identified the state-of-the-art statistical methodology currently employed when using individual-specific networks for prediction in medicine and applied health research. We found a wide range of applications and methodological concepts in our review. We collated the key concepts identified across the 148 included studies considering three main aspects of modelling with individual-specific networks: (1) individual-specific network inference, (2) extraction of graph-theoretical features, and (3) prediction modelling. Within each of these aspects, there is considerable methodological heterogeneity in the implementation, use, areas of application, and reporting. However, all approaches outlined in this work are in principle generalizable to any field of research and may be suitable to answer various prognostic or diagnostic research questions in medicine.

Individual-specific networks were frequently constructed by evaluating correlations between repeated measurements of pairs of variables (e.g. time-series data of two brain regions). Here we identified two main approaches based on bivariate and partial correlation analysis, some variants thereof, and some further approaches that were often tied to the process of data acquisition itself. Furthermore, the recently proposed visibility graphs offer a flexible approach to individual-specific network inference in relation to time series data. However, sometimes several time series are available per individual so that the individual-specific network is ambiguous, which leads to a further layer of complexity. In the absence of repeated measurements per variable, two novel and promising approaches were LOONC and differential perturbation. Despite their flexibility and ease of application to continuous independent variables, a big challenge in the latter two approaches remains the considerable computational burden, in particular in high-dimensional data settings, due to the repeated computation of the augmented network for each individual [57].

In the pursuit to separate ‘real’ from ‘spurious’ connections, in addition to the need for a reduced computational burden, network sparsification has become an important aspect of network analysis. However, sparsification not only depends on the selected technique but, more importantly, also on the chosen threshold, and hence, often multiple thresholds were employed to reduce the impact of a possibly flawed choice, yielding a sequence of networks per individual with varying edge weights. Various approaches were then applied to deal with the sequence of networks; numerical integration or averaging were the most popular approaches together with a threshold selection yielding the best AUROC. Although the majority of studies refrained from using a single, arbitrary threshold value, multiple edge weighting schemes and sparsification strategies were seldom guided by model fit. In addition, sensitivity analyses evaluating the impact of threshold choice on predictive performance were expected but hardly found.

For the extraction of graph-theoretical features, we found a set of global and local features (e.g. see Table 2) that were used in many studies across research fields. For the most part, the clustering coefficient, the characteristic path length and the edge weights were examined in the search for potential biomarkers of the outcome of interest. Furthermore, the extraction of graph-theoretical features did not follow a deliberate process but often consisted of a greedy collection of network characteristics i.e. the computation and outcome-associated investigation of as many graph-theoretical variables as possible.

Despite the multiplicity of identified methodological approaches for individual-specific network inference across fields of application, the number of studies that proposed models actually intended to provide clinical outcome prediction for future individuals was quite limited, and most models were estimated to provide a proof-of-concept that graph-theoretical features may in principle be useful for outcome prediction. The lack of deployable clinical prediction models could be either a consequence of the fundamental challenges in network inference methods shared by all areas of application, in particular concerning the lack of a gold standard for network construction and sparsification, or of the general unawareness in how individual-specific networks can be exploited for prediction.

Through the systematic collation of the identified analytical approaches, we found some areas interesting for future research and which may help to reduce some of the arbitrariness of some analytical choices.

First, more research is needed to reduce the computational burden related to the construction and analysis of relatively large and dense networks and the inherent computational complexity of graph metric computation. Large omics studies generate substantial amounts of data which can lead to major computational difficulties in network inference and further analysis, if each node in the network represents a single variable. In particular, LOONC and differential perturbation approaches would suffer in such a data setting due to the computational burden caused by the repeated network computation for each individual in the study cohort. One possible strategy for reducing network complexity and facilitating network analysis and feature extraction could be node aggregation over groups of connected, non-independent nodes (modules). In this sense, variables could be combined as modules either through unsupervised clustering algorithms or through biological background knowledge, so that each node represents a group of independent variables and no longer a single variable.

Second, network sparsification and multivariable model estimation could be linked more closely by guiding the search for a suitable threshold or the integration over several thresholds by the model fit. Multiple thresholding yields a set of sequential graph-theoretical estimates of the networks that are computed over a fine grid on a continuous domain using incremental steps between threshold values (e.g. grid searching). Ideas from functional data analysis could be transferred to the area of modelling with individual-specific networks. Briefly, instead of choosing a threshold that provides univariably optimal prediction performance, or integration over multiple pre-specified thresholds with equal weights of each threshold, one could interpret the individual-specific sequence of graph-theoretical features corresponding to the set of sparsified networks ${\mathcal{G}}_s:= {\left\{{G}_{s,{\tau}_k}\right\}}_{k=1,\dots, \mathrm{T}}$ as a functional data predictor. Then, one may define a flexible weighting function of *τ*_*k*_, *f*(*τ*_*k*_), through outcome-guided calibration to optimize clinical prediction. Consequently, such an analysis would also yield an estimate of the relative importance of different thresholds for network sparsification. Alternatively, carefully conducted sensitivity analyses would allow evaluating to what extent the reported results depended on the choice of the selected threshold value in particular for studies continuing the search for the optimal threshold parameter by univariate analysis.

Further, the comparison of networks of varying sizes and edge densities can impose issues for prediction modelling since some graph-theoretical features are confounded by them [80]. The inclusion of these two features regardless of ‘significance’ may reduce the magnitude of bias, and improve prediction performance and explainability of such models [105]. Omission of these confounding variables could mask the actual effects of interest in model explanation. Generally, graph-theoretic features are inherently associated with each other, and more research is needed to better understand these associations.

In contrast, a reoccurring problem of multivariable model building was found in the evaluated studies: “univariable prefiltering” of variables in which only variables with a statistically significant association with the outcome are included in the model [106, 107]. However, a *p*-value above the statistical significance threshold of 5% is not sufficient evidence for the lack of an effect of the independent variable [108]. The popularity of prefiltering across the evaluated studies can presumably be traced back to the greedy collection of graph-theoretical features or to a disproportional number of local graph features that were obtained relative to sample size. Since the actual goal of univariable preselection was often a considerable reduction of the number of independent variables proportional to the sample size to seemingly avoid overfit, defining a minimum basic set of features (MBSF) to investigate may be beneficial when network analysis is employed for prediction. The identification of such an MBSF, however, is not an easy task and may require investigations across a range of applications embedded in the respective research fields.

Lastly, future studies should investigate the added benefit of graph-theoretical features in addition to clinical variables so as not to examine their clinical utility separately from traditional variables and hence, improve existing clinical prediction models. We have seen that graph-theoretical variables were mostly examined independently, which was partly due to the relatively low samples across the evaluated studies but also due to the dominant preference of algorithmic classification approaches with local graph-theoretical features, where clinical information was largely ignored. In some studies, in which graph-theoretical features were examined together with clinical variables, these even turned out to be stronger predictors than the traditional set of clinical variables. It remains elusive if this could be explained by publication bias or demonstrates the clinical relevance of graph-theoretical features.

Research on the aforementioned points is essential to establish a state-of-the-art and to provide more evidence-based guidance in using individual-specific networks for the prediction of clinical outcomes to applied researchers. Despite the identified aspects for future research, our scoping review is subject to some limitations. First, we may have missed some relevant applications due to the lack of standardized terminology to describe the intersection of network analysis (in particular approaches to construct individual-specific networks) and prediction modelling but also because of the ambiguity of the term ‘network’. Secondly, by limiting our study to applications in medicine and health research, we may not have captured studies that employed individual-specific networks in which the individuals do not represent patients but other individual entities. Thirdly, since the screening of 4988 studies and extracting data for the 148 articles included in this review was laborious, some time passed between identification of studies and completion of data extraction. Nevertheless, we decided not to update our search to include more recent articles (i.e. published from August 2020 onwards) because we do not believe that there were substantial changes in practice in the intervening time. Last but not least, this review focused on the use of individual-specific network analysis for prediction which is why existing refinements of the presented methods for network construction and extraction of graph-theoretic ones might not have passed the inclusion criteria of our search strategy.

At this juncture, it is important to emphasize that we have not assessed the quality of included studies and did not perform a risk of bias assessment. This was done in agreement with the general guidelines on conducting a scoping review. Consequently, our review is unable to make definitive recommendations for practice but rather describes the current methodological practice and possible areas of future research [7, 109].

## Conclusion

Network analysis offers a flexible tool for personalized medicine, hence prediction with individual-specific networks is an emerging field full of potential for future research. The application in clinical research is still in its infancy but our findings can strengthen the methodological conduct to incorporate individual-specific network analysis in predictive tasks. The framework still requires further refinement, and research must cover statistical, computational and application-specific aspects. In addition to methodological advances, comparative studies of proposed methodologies are needed to understand how methods compare and which method works best in a specific setting. This may eventually lead to establishing a state-of-the-art in this novel and fascinating scientific arena located at the cross-section of statistics, computer science and medicine.

## Supplementary Information

**Additional file 1.** Preferred Reporting Items for Systematic reviews and Meta-Analyses extension for Scoping Reviews (PRISMA-ScR) Checklist

**Additional file 2: Supplementary Material S1.** Detailed Methods. **Table S1.** Keyword sets used in the search strategy across the databases for article extraction. **Figure S1.** Year of publication of the identified studies. **Table S2.** Scale of the graph-theoretical features used as candidate predictors stratified by the area of application.

**Additional file 3. Supplementary Material S2.** Questionnaire for full-text screening

## Data Availability

Not applicable.

## Abbreviations

AUC: Area under the curve
A: Assortativity
BC: Betweenness centrality
BOLD: Blood oxygenation level-dependent
CC: Clustering coefficient
CPL: Characteristic path length
DAG: Directed acyclic graph;
Dg: Degree
Ds: Density
EEG: Electroencephalography
EW: Edge weight
GE: Global efficiency
HRV: Heart rate variability
IQR: Interquartile range
LASSO: Least absolute shrinkage and selection operator
LE: Local efficiency
LOONC: Leave-one-out network construction
LOOCV: Leave-one-out cross-validation
M: Modularity
MBSF: Minimum basic set of features
RFE: Recursive feature elimination
SD: Standard deviation
SVM: Support vector machine
SWI: Small world index
ROI: Region of interest
VAR: Vector autoregressive
