# Supplementary material for: Individual-specific networks for prediction modelling – A scoping review of methods
Source: BMC Med Res Methodol. 2022 Mar 6;22:62. doi: 10.1186/s12874-022-01544-6 (PMC8898441; doi:10.1186/s12874-022-01544-6)
Supplement: Supplementary file 2 — Additional file 2: Supplementary Material S1. Detailed Methods. Table S1. Keyword sets used in the search strategy across the databases for article extraction. Figure S1. Year of publication of the identified studies. Table S2. Scale of the graph-theoretical features used as candidate predictors stratified by the area of application. [file 12874_2022_1544_MOESM2_ESM.docx]

**Individual-specific networks for prediction modelling – A scoping review of methods**

Supplementary Material S1 – Detailed Methods

# ADDITIONAL METHODS

## Search Strategy

We conducted a scoping review to assess the use of graph-theoretical features from individual-specific networks in prediction modelling and various field of application in medicine and biology. Relevant studies were identified by searching PubMed, Scopus and Embase published in journals after January 1^st^, 2000 up until August 31, 2020, using a pairwise combination of two main sets of keywords: (1) words indicative of graph theory (e.g. n*etwork*, *graph-theory*) and (2) words indicative of prediction modelling as well as a set of exclusion keywords to increase the proportion of relevant articles. The keyword fields, which were investigated were the title and the abstract. The two main sets of keywords were used for all databases. Excluding keywords in the query syntax were varied in between the databases depending on the abundance of articles found.

The two main keyword sets and the respective terms used for the search strategy are listed in Supplementary Table 1.

Table S1. Keyword sets used in the search strategy across the databases for article extraction

| **Keyword Set** | **Terms** | **Position** |
| --- | --- | --- |
| **Network** | Network parameters  OR network variables  OR network attributes  OR network traits  OR network features  OR network characteristics  OR graph-theory  OR graph-theoretic | Title/Abstract |
| **Prediction Modelling** | Prognosis  OR predictive  OR prediction  OR predict  OR predicting  OR predictor  OR forecast  OR forecasting | Title/Abstract |
| **Exclusion Keywords** | NOT neural network  NOT neural networks  NOT artificial networks  NOT learning network  NOT learning networks  NOT adversarial network  NOT adversarial networks  NOT link prediction  NOT signal network  NOT sensor network | Title/Abstract |

As an example, the search for the PubMed database can be replicated by the following link:

[https://pubmed.ncbi.nlm.nih.gov/?term=%28%22graphtheory%22%5BTitle%2FAbstract%5D+OR+%22graphtheoretic%22%5BTitle%2FAbstract%5D+OR+%22network+parameters%22%5BTitle%2FAbstract%5D+OR+%22network+variables%22%5BTitle%2FAbstract%5D+OR+%22network+attributes%22%5BTitle%2FAbstract%5D+OR+%22network+traits%22%5BTitle%2FAbstract%5D+OR+%22network+features%22%5BTitle%2FAbstract%5D+OR+%22network+characteristics%22%5BTitle%2FAbstract%5D%29+AND+%28%22predictive%22%5BTitle%2FAbstract%5D+OR+%22predictive%22%5BTitle%2FAbstract%5D+OR+%22prediction%22%5BTitle%2FAbstract%5D+OR+%22prognosis%22%5BTitle%2FAbstract%5D+OR+%22predict%22%5BTitle%2FAbstract%5D+OR+%22prediction%22%5BTitle%2FAbstract%5D+OR+forecast%5BTitle%2FAbstract%5D+OR+%22forecasting%22%5BTitle%2FAbstract%5D%29+NOT+%28%22neural+network%22%5BTitle%2FAbstract%5D+OR+%22neural+networks%22%5BTitle%2FAbstract%5D+OR+%22artificial+networks%22%5BTitle%2FAbstract%5D+OR+%22learning+network%22%5BTitle%2FAbstract%5D+OR+%22learning+networks%22%5BTitle%2FAbstract%5D+OR+%22adversarial+network%22%5BTitle%2FAbstract%5D+OR+%22adversarial+networks%22%5BTitle%2FAbstract%5D+OR+%22link+prediction%22%5BTitle%2FAbstract%5D+OR+%22signal+network%22%5BTitle%2FAbstract%5D+OR+%22sensor+network%22%5BTitle%2FAbstract%5D%29&filter=dates.2000%2F1%2F1-2020%2F8%2F31](https://pubmed.ncbi.nlm.nih.gov/?term=%28%22graphtheory%22%5BTitle%2FAbstract%5D+OR+%22graphtheoretic%22%5BTitle%2FAbstract%5D+OR+%22network+parameters%22%5BTitle%2FAbstract%5D+OR+%22network+variables%22%5BTitle%2FAbstract%5D+OR+%22network+attributes%22%5BTitle%2FAbstract%5D+OR+%22network+traits%22%5BTitle%2FAbstract%5D+OR+%22network+features%22%5BTitle%2FAbstract%5D+OR+%22network+characteristics%22%5BTitle%2FAbstract%5D%29+AND+%28%22prognostic%22%5BTitle%2FAbstract%5D+OR+%22predictive%22%5BTitle%2FAbstract%5D+OR+%22prediction%22%5BTitle%2FAbstract%5D+OR+%22prognosis%22%5BTitle%2FAbstract%5D+OR+%22predict%22%5BTitle%2FAbstract%5D+OR+%22prediction%22%5BTitle%2FAbstract%5D+OR+forecast%5BTitle%2FAbstract%5D+OR+%22forecasting%22%5BTitle%2FAbstract%5D%29+NOT+%28%22neural+network%22%5BTitle%2FAbstract%5D+OR+%22neural+networks%22%5BTitle%2FAbstract%5D+OR+%22artificial+networks%22%5BTitle%2FAbstract%5D+OR+%22learning+network%22%5BTitle%2FAbstract%5D+OR+%22learning+networks%22%5BTitle%2FAbstract%5D+OR+%22adversarial+network%22%5BTitle%2FAbstract%5D+OR+%22adversarial+networks%22%5BTitle%2FAbstract%5D+OR+%22link+prediction%22%5BTitle%2FAbstract%5D+OR+%22signal+network%22%5BTitle%2FAbstract%5D+OR+%22sensor+network%22%5BTitle%2FAbstract%5D%29&filter=dates.2000%2F1%2F1-2020%2F8%2F31)

## Inclusion and Exclusion Criteria

Article Inclusion:

1. The description of a method to include individual-specific networks or their graph-theoretical attributes as independent predictors in prediction modelling.
2. The application of a method which includes individual-specific networks or their graph-theoretical attributes as independent predictors in prediction modelling.
3. An overview of several methods to include individual-specific networks or their graph-theoretical attributes as independent predictors in prediction modelling (e.g. review article, commentary).
4. The comparison of several methods (e.g. via simulation studies) to include individual-specific networks or their graph-theoretical attributes as independent predictors in prediction modelling

Article Exclusion:

1. The article is a duplicate (arising from e.g. using two different search engines)
2. The article focuses on group-level (multi-individual) networks derived from aggregated data and not on single-individual networks.
3. The evaluation of the network characteristics is performed for descriptive purposes of the network itself only but without an outcome of interest.
4. The article focuses on using the networks or their attributes to predict further links, node interactions or dynamic change in the networks (i.e. a graph-theoretical feature constitutes the dependent variable and not the independent predictor).
5. The term network is used in another context (e.g. to describe a complex pattern, interlinked components) but neither the network nor its graph-theoretical attributes are used in a statistical model.

## Selection of Articles

Articles not written in English were excluded. Identified articles by the search strategy stated above were first assessed for eligibility based on the titles. To ensure objectivity of the first reviewer with regard to the inclusion and exclusion of articles, a random subset of articles in each screening phase (250 articles for title screening, 50 for abstract screening, 25 for full-text assessment) was extracted at random and assessed by three additional independent reviewers (GH, FM, MS). Any inconsistencies were discussed and resolved to reach a general consensus. Studies for which we were not able to discern the presumed relevance for the review from the title or abstract were included for further evaluation. After screening of the abstracts, all identified articles were read in full-text and manuscripts which did not focus on prediction modelling with graph-theoretical features derived from individual-specific networks were excluded from the data extraction process. Furthermore, articles were selected based on the inclusion criteria or excluded based on the exclusion criteria prespecified in the protocol of the scoping review. References of the selected articles were checked for articles fulfilling the inclusion criteria of the scoping review. Articles from the references list of selected articles that present a review were only included if the article differentiates itself methodologically from the other studies. In addition, we included articles identified through a manual search of the literature. Title and abstract screening were performed using the package ‘*revtools*’ in the programming language R (Version 4.0.2).

## Data Extraction

The scoping review adhered to an a priori protocol and followed the reporting guidelines outlined in the PRISMA extension for scoping reviews (PRISMA-ScR) developed for systematic evidence synthesis from a body of literature heterogeneous in methodology and its fields of application [1]. The PRISMA-ScR checklist corresponding to the review is presented in the Supplementary Material. Further, we considered aspects of Transparent Reporting of a multivariable prediction model for Individual Prognosis Or Diagnosis (TRIPOD) Initiative [2]. However, risk of bias assessment does not constitute a component of a scoping review. Key features of data extraction from the full-text assessment focused on 1) general information on the study (e.g. study objective, study cohort, research discipline, availability of code and data), 2) network analysis such as network construction (e.g. size, setup, network sparsification) and the computed set of graph-theoretical attributes from the individual-specific networks (e.g. local or global, normalization) and 3) the multivariable outcome model including model building (e.g. the aim of the model, study endpoint, sample size, method, candidate predictors, variable selection) and model validation (e.g. internal, external or apparent, discrimination and calibration) and 4) the conduct of the simulation study where appropriate. Data extraction of full-text was conducted in the online survey software Google Forms in which a questionnaire was created with access only for the involved reviewers upon invite from the creator.

However, the search strategy described also has its limitations. The term "network" in particular is a broad and widely used term in a wide variety of contexts. Some exclusion terms were defined to limit the resulting studies, but also means that relevant studies could have been affected by this as well. Furthermore, through cooperation with other independent reviewers (GH, FM, MS), an attempt was made to guarantee the objectivity of the main authors, but the selection and exclusion of papers is still to a certain extent subjective.

# ADDITIONAL RESULTS

## General Findings

Figure S1 presents the distribution of identified articles with respect to year of publication. Apart from the exceptional year 2018 in the left panel of Figure S1, we suspect an ongoing increase in interest in individual-specific networks for the aim of personalized prediction.


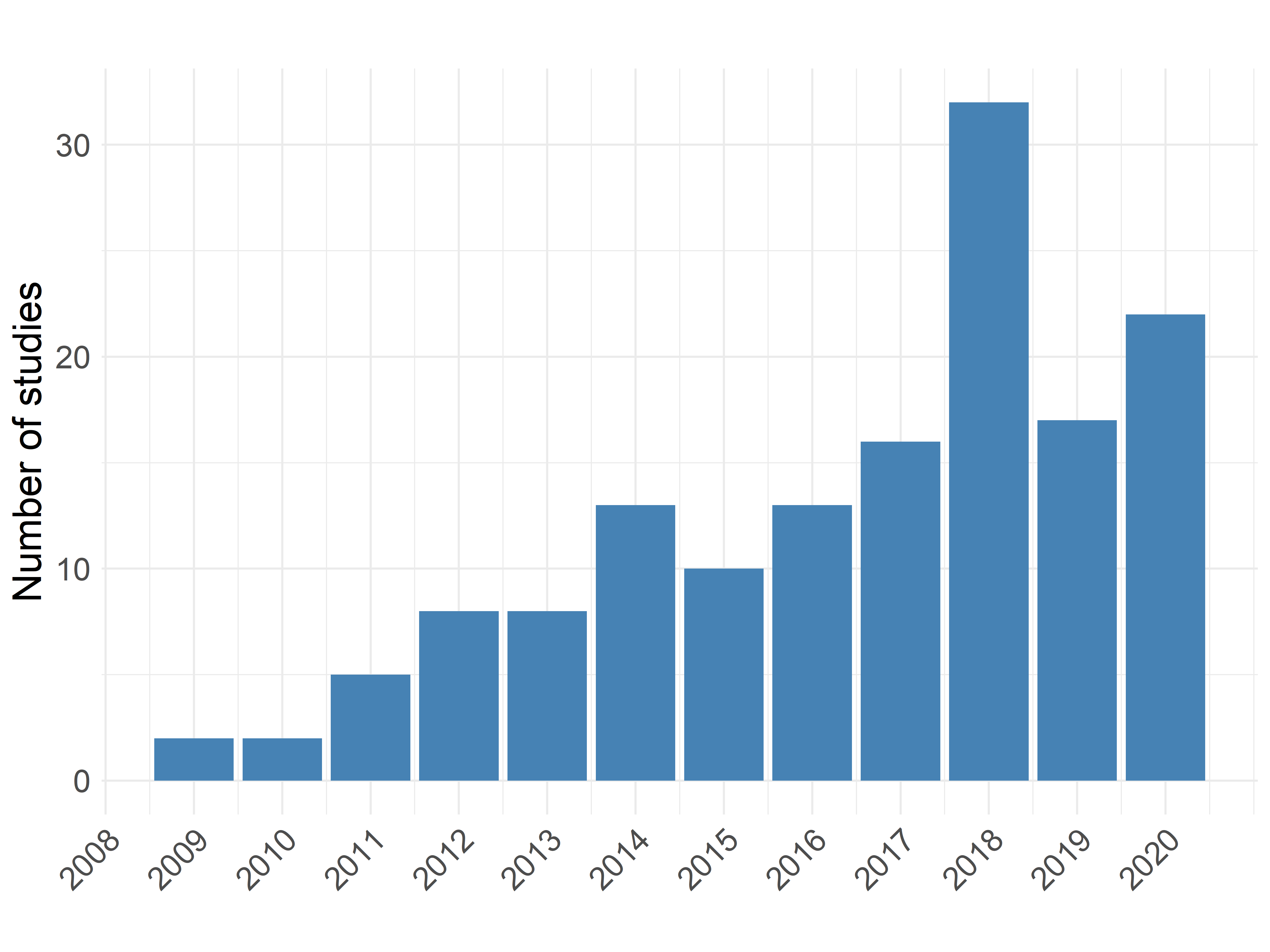


Figure S1. Year of publication of the identified studies

## Domain-specific findings of the scoping review

Graph-theoretical analysis of (multiple) neuroimaging data modalities has rapidly found application in clinical neuroscience to identify brain network-based biomarkers to predict and diagnose neurological health outcomes such as mental illnesses [3], neurological diseases [4-6], brain injuries [7, 8] or cognitive functioning [9]. Disturbed brain organization and topology have been shown to be indicative of neurological issues and hence make graph-theoretical features well-founded candidates for potential diagnostic biomarkers. For instance, patients with mild cognitive impairment (MCI) had grey matter network measures that were indicative of a more random network topology at the time of first visit, showed a steeper rate of decline in cognitive functioning [4, 10]. A majority of the neurological studies stated small sample size as a limitation of the study which makes statistical power a concern and affects model generalization to unseen data [11-20] but also in other areas small sample size seemed to be an issue [21]. Network analysis in neurological studies has already shown that the brain structure of healthy individuals has certain properties such as balanced integration and segregation, while patients with neurological disorders such as epilepsy approach a random network structure [11].

Table S2. Scale of the graph-theoretical features used as candidate predictors stratified by the area of application

|  | **Graph-theoretical scale** | | |
| --- | --- | --- | --- |
| **Area of application** | Global | Local | Both |
| Genomics | 0 | 3 | 0 |
| Neurology | 12 | 34 | 29 |
| Pathopsychology | 2 | 1 | 3 |
| Pathology | 0 | 0 | 1 |

Psychological studies were mostly mainly interested in determining the progression and outcome of early-stage psychotic symptoms given self-administered questionnaires for the purpose of routine screening and quantifying symptom severity [22]. To investigate the inherent daily dynamics of self-reported symptoms, vector autoregressive models (VARs) were conducted and associations between symptoms represented the strength of connection within the individualized networks. A highly relevant feature for pathopsychology was centrality to identify highly influential nodes representing centrally connected symptoms or root causes which can then be targeted by therapeutic strategies – so called early warning signals [23]. [21] conducted a proof-of-concept study to exhibit the benefit of adding graph-theoretical features in addition to patient information for the prediction of dropout in psychological treatments and found varying network structures for patient completing and those who drop out of treatment.

For cancer research, the individual-specific gene networks were employed by [24] to capture the heterogeneity of gene-gene interaction networks of breast cancer samples across individuals since according to them, network-based methods have shown more robust and efficient behaviour than single-gene methods for noisy high-throughput data. To identify local gene pair biomarkers for personalized breast cancer prognosis, a Cox proportional hazards regression model was used with prior univariate variable selection and the least absolute shrinkage and selection operator (LASSO) reducing the number of candidate features from 46,916 to 272 final gene pairs. The individualized perturbation networks were further assessed to determine tipping points of complex disease (cancer) prior to disease deterioration by assessing a composite network score based on the edge weights at every available time point [25, 26] or for exploratory analysis of predictive gene pairs in cancer [27]. Huang et al. [28] generated perturbed individual-specific gene networks and was able to reach a classification accuracy of 100% using hierarchical clustering based on in-between network distance. In particular, in the network distance matrix $D=\left( d_{ij} \right)_{N\times N}$where $N$ denotes the number of individuals, each cell value corresponds to the distance between two sample-specific networks defined as the ratio of the number of overlapping edges and the total number of edges.

Studies outside of neurology, pathopyschology and cancer research aimed for instance at the diagnosis of muscle disease with a large set of graph-theoretical features (N=44; total number of features N=82) obtained from the individual-specific muscle fibre networks using neural networks [29] or were focused on individual-specific networks of the heart rate variability to delineate between patients with obstructive sleep apnoea and controls [30].

**References**

1. Tricco AC, Lillie E, Zarin W, O'Brien KK, Colquhoun H, Levac D, et al. PRISMA extension for scoping reviews (PRISMA-ScR): checklist and explanation. Annals of Internal Medicine. 2018;169(7):467-73.

2. Collins GS, Reitsma JB, Altman DG, Moons KG. Transparent Reporting of a Multivariable Prediction Model for Individual Prognosis or Diagnosis (TRIPOD) The TRIPOD Statement. Circulation. 2015;131(2):211-9.

3. Rashid B, Calhoun V. Towards a brain‐based predictome of mental illness. Human Brain Mapping. 2020;41(12):3468-535.

4. Tijms BM, Wink AM, de Haan W, van der Flier WM, Stam CJ, Scheltens P, et al. Alzheimer's disease: connecting findings from graph theoretical studies of brain networks. Neurobiology of Aging. 2013;34(8):2023-36.

5. Bernhardt BC, Bonilha L, Gross DW. Network analysis for a network disorder: the emerging role of graph theory in the study of epilepsy. Epilepsy & Behavior. 2015;50:162-70.

6. Guye M, Bettus G, Bartolomei F, Cozzone PJ. Graph theoretical analysis of structural and functional connectivity MRI in normal and pathological brain networks. Magnetic Resonance Materials in Physics, Biology and Medicine. 2010;23(5):409-21.

7. Imms P, Clemente A, Cook M, D’Souza W, Wilson PH, Jones DK, et al. The structural connectome in traumatic brain injury: A meta-analysis of graph metrics. Neuroscience & Biobehavioral Reviews. 2019;99:128-37.

8. Caeyenberghs K, Verhelst H, Clemente A, Wilson PH. Mapping the functional connectome in traumatic brain injury: What can graph metrics tell us? NeuroImage. 2017;160:113-23.

9. Yamashita M, Kawato M, Imamizu H. Predicting learning plateau of working memory from whole-brain intrinsic network connectivity patterns. Scientific Reports. 2015;5(1):1-8.

10. Dicks E, Tijms BM, Ten Kate M, Gouw AA, Benedictus MR, Teunissen CE, et al. Gray matter network measures are associated with cognitive decline in mild cognitive impairment. Neurobiology of Aging. 2018;61:198-206.

11. Babajani-Feremi A, Noorizadeh N, Mudigoudar B, Wheless JW. Predicting seizure outcome of vagus nerve stimulation using MEG-based network topology. NeuroImage: Clinical. 2018;19:990-9.

12. Batalle D, Eixarch E, Figueras F, Muñoz-Moreno E, Bargallo N, Illa M, et al. Altered small-world topology of structural brain networks in infants with intrauterine growth restriction and its association with later neurodevelopmental outcome. NeuroImage. 2012;60(2):1352-66.

13. Jie B, Zhang D, Wee CY, Shen D. Topological graph kernel on multiple thresholded functional connectivity networks for mild cognitive impairment classification. Human Brain Mapping. 2014;35(7):2876-97.

14. Khazaee A, Ebrahimzadeh A, Babajani-Feremi A. Identifying patients with Alzheimer’s disease using resting-state fMRI and graph theory. Clinical Neurophysiology. 2015;126(11):2132-41.

15. Sun Y, Bi Q, Wang X, Hu X, Li H, Li X, et al. Prediction of conversion from amnestic mild cognitive impairment to Alzheimer's disease based on the brain structural connectome. Frontiers in Neurology. 2019;9:1178.

16. Wee C-Y, Yap P-T, Zhang D, Denny K, Browndyke JN, Potter GG, et al. Identification of MCI individuals using structural and functional connectivity networks. NeuroImage. 2012;59(3):2045-56.

17. Wee C-Y, Yap P-T, Li W, Denny K, Browndyke JN, Potter GG, et al. Enriched white matter connectivity networks for accurate identification of MCI patients. NeuroImage. 2011;54(3):1812-22.

18. Du J, Wang Y, Zhi N, Geng J, Cao W, Yu L, et al. Structural brain network measures are superior to vascular burden scores in predicting early cognitive impairment in post stroke patients with small vessel disease. NeuroImage: Clinical. 2019;22:101712.

19. Sen B, Bernstein GA, Mueller BA, Cullen KR, Parhi KK. Sub-graph entropy based network approaches for classifying adolescent obsessive-compulsive disorder from resting-state functional MRI. NeuroImage: Clinical. 2020;26:102208.

20. Liu L, Zhang H, Wu J, Yu Z, Chen X, Rekik I, et al. Overall survival time prediction for high-grade glioma patients based on large-scale brain functional networks. Brain Imaging Behaviour. 2019;13(5):1333-51.

21. Lutz W, Schwartz B, Hofmann SG, Fisher AJ, Husen K, Rubel JA. Using network analysis for the prediction of treatment dropout in patients with mood and anxiety disorders: A methodological proof-of-concept study. Scientific Reports. 2018;8(1):1-9.

22. Booij SH, Wichers M, De Jonge P, Sytema S, Van Os J, Wunderink L, et al. Study protocol for a prospective cohort study examining the predictive potential of dynamic symptom networks for the onset and progression of psychosis: the Mapping Individual Routes of Risk and Resilience (Mirorr) study. BMJ Open. 2018;8(1).

23. Fried EI, van Borkulo CD, Cramer AO, Boschloo L, Schoevers RA, Borsboom D. Mental disorders as networks of problems: a review of recent insights. Social Psychiatry and Psychiatric Epidemiology. 2017;52(1):1-10.

24. Zhu K, Pian C, Xiang Q, Liu X, Chen Y. Personalized analysis of breast cancer using sample-specific networks. PeerJ. 2020;8:e9161.

25. Liu X, Chang X, Leng S, Tang H, Aihara K, Chen L. Detection for disease tipping points by landscape dynamic network biomarkers. National Science Review. 2019;6(4):775-85.

26. Yu X, Zhang J, Sun S, Zhou X, Zeng T, Chen L. Individual-specific edge-network analysis for disease prediction. Nucleic Acids Research. 2017;45(20):e170-e.

27. Park B, Lee W, Park I, Han K. Finding prognostic gene pairs for cancer from patient-specific gene networks. BMC Medical Genomics. 2019;12(8):1-14.

28. Huang Y, Chang X, Zhang Y, Chen L, Liu X. Disease characterization using a partial correlation-based sample-specific network. Briefings in Bioinformatics. 2020.

29. Sáez A, Rivas E, Montero-Sánchez A, Paradas C, Acha B, Pascual A, et al. Quantifiable diagnosis of muscular dystrophies and neurogenic atrophies through network analysis. BMC Medicine. 2013;11(1):1-11.

30. Dong Z, Li X, Chen W. Frequency network analysis of heart rate variability for obstructive apnea patient detection. IEEE Journal of Biomedical and Health Informatics. 2017;22(6):1895-905.
