# Supplementary material for: Individual-specific networks for prediction modelling – A scoping review of methods
Source: BMC Med Res Methodol. 2022 Mar 6;22:62. doi: 10.1186/s12874-022-01544-6 (PMC8898441; doi:10.1186/s12874-022-01544-6)
Supplement: Supplementary file 3 — Additional file 3. Supplementary Material S2. Questionnaire for full-text screening [file 12874_2022_1544_MOESM3_ESM.docx]

**Individual-specific networks for prediction modelling – A scoping review of methods**

Supplementary Material S2 – Questionnaire for full-text screening

**General Information**

1. E-Mail-Address *
2. Title of the paper *
3. DOI of the paper *
4. Authors of the paper *
5. Journal *
6. Year of publication *
7. In what subject-matter is the publication embedded in? (research discipline) *

e.g. Neurology, Psychology, Social Sciences, Oncology, ...

1. Aim(s) of the paper *

**Inclusion/Exclusion Criteria**

1. The following exclusion criterion holds: The networks and/or graph-theoretic attributes described in the article are *

individual-specific and the article fulfills the inclusion criteria stated in the SR protocol. *(Continue with question 10)*

not individual-specific. *(Continue with question 92)*

used only for descriptive purposes without a focus on an outcome of interest. *(Continue with question 92)*

used to predict further network links, node interactions or the dynamic change of the networks. *(Continue with question 92)*

used in another context (e.g. a complex pattern, interlinked structure or social network) *(Continue with question 92)*

the physical phenomena are modeled by ODEs or PDEs *(Continue with question 92)*

Sonstiges:

**Study or Commentary?**

1. Study type of the article *

The article conducts individual-specific network analysis to improve prognostic modelling. *(Continue with question 24)*

The article presents the idea or concept of individual-specific network analysis in its field *(Continue with question 11)*

Concept of individual-specific networks to improve prognostic models

This section focuses on articles which state the idea or concept of individual-specific networks in its respective scientific field but do not perform some sort of quantitative analysis such as a data analysis or simulation study.

1. The article... *

presents individual-specific networks as a novel approach in its specific field. presents individual-specific networks as a well-known approach in its specific field. presents individual-specific networks as a rarely used approach in its specific field.

Other:

1. The article is a *

Commentary Review

Systematic Review Meta-Analysis

1. What is the suggested individual-specific network about? *
2. How are the vertices defined? *
3. How are the edges defined? *
4. Does the article suggest using the networks and/or their attributes in a prognostic context? *

No Yes

Other:

1. If the approach is considered in a prognostic context: What is the outcome of interest?
2. If the approach is considered in a prognostic context: What type is the outcome?

Binary Continuous Time-to-event Ordinal Nominal

Rate

Other:

1. If the approach is considered in a prognostic context: What methods are proposed to build a prognostic model?
2. Additional information
3. Were any limitations of the approach stated? *
4. Summarize the conclusion of the paper *
5. State the future perspectives of the approach if stated by the author(s) *

*(Continue with question 86)*

**Individual-specific networks analysis**

1. Description of the individual-specific networks *

i.e. background, aim

1. Population of interest *
2. Data acquisition for the networks

Clinical-epidemiological features Electroencephalography (EEG)

Functional magnetic resonance imaging (fMRI) Magnetic resonance imaging (MRI)

Diffusion Tensor Imaging (DTI)

Ecological Momentary Assessments (EMA) RNA-sequencing

Other:

1. Sample size *
2. Number of study subjects with the outcome *

i.e. Cases= ..., Controls=...

All Unclear

Other:

1. Study type *

Prospective cohort Retrospective cohort Nested case-control Case-control

Randomized trial participants Registry data

Unclear

Other:

1. Specify the network construction approach

Biologically informed Pearson correlation

Partial correlation Distributional similarity Coherence-based

Phase lag index

Vector autoregressive model Network perturbation analysis Leave-one-out approach

Other:

1. How are the vertices defined? *

If multiple networks were considered with varying vertex definitions, specify each definition separately by (1) ... (2)....

Brain regions of interest Genes

EEG electrodes Psychotic symptoms MEG electrodes

Other:

1. How are the edges defined? (short version) *

If multiple networks were considered with varying edge conditions, specify each condition separately by (1) ... (2) ....

Pearson correlation of time series

Pearson correlation of cortical thickness

Pairwise connectivity strength between electrodes

Partial correlation

Vector autoregressive models

Fiber pathways

Phase lag index

Other:

1. If an adjacency matrix was set up, how was it derived? (long version)

If multiple networks were considered with varying definitions of adjacency matrices, specify each definition separately by (1) ... (2) ....

1. Which network sparsification approach was considered? *

(Binarization can be combined with weight-based or density-based thresholding)

No network sparsification Binarization

Weight-based thresholding/Trimming Proportional/Density-based thresholding Consensus thresholding

Univariate hypothesis testing for significance of edges Other:

1. Are multiple threshold cutoffs for network sparsification considered? *

Yes No

Unclear

Other:

1. If multiple thresholds: Was the further analysis conducted considering only one of the thresholds? *

Thresholding not applied to the networks

Yes, only one threshold was used

Yes, but the chosen threshold was determined (e.g. AUC)

Yes, the center of the thresholding range was used

No, network parameters were obtained for different thresholding cutoffs

Unclear

Other:

1. The final analysis was carried out with the graph-theoretical features *

obtained from the weighted networks.

but without specifying how it was done in relation to thresholding.

from the networks under a single threshold cutoff.

averaged from the range of thresholded networks for one individual.

from the individual's network yielding the best AUC.

calculated from networks under different thresholding cutoffs.

Unclear

Other:

1. Further describe the network sparsification process if needed
2. The individual-specific networks were computed from information taken

at baseline

at one follow-up after baseline but before the outcome was known simultaneously with the complete data collection (including outcome)

at one follow-up after the outcome was already known

Unclear Not stated

Other:

1. How many vertices and edges do the individual-specific networks contain on average? *

Fill in as Vertices=XXX, Nodes=XXX

Not stated Other:

1. Are multiple types of vertices or edges considered in the network? *

e.g. multiple conditions for connectivity are investigated and multiple node sets are considered

Multiple types of edges

Multiple types of vertices

Multiples types of vertices and edges

No

Unclear

Other:

1. Are the individual-specific networks weighted? *

Weighted edges Weighted vertices

Weighted vertices and edges Weighted and binarized edges

No

Unclear

Other:

1. If the networks are weighted, how are the weights defined?
2. Are the individual-specific networks directed or undirected? *

Undirected Directed Unclear

Other:

1. Are dynamic individual-specific networks considered? *

i.e. the temporal change of the individual-specific networks is assessed

Yes No

Other:

1. What graph-theoretic attributes are used to describe the network? *

Assortativity coefficient

Authority

Characteristic path length Betweenness centrality Clustering coefficient Closeness centrality

Diameter

Degree Density

Nodal efficiency Nodal strength

Edge weight

Eigenvector centrality Global efficiency

Largest component size Local efficiency Modularity

Network size

Normalized characteristic path length Normalized clustering coefficient

PageRank index

Participation coefficient Small-world index Transitivity

Other:

1. Were local and/or global parameters of the individual-specific networks computed?

Global Local

Global and local Unclear

1. Number of global features assessed *
2. Number of local features assessed *
3. Were some of the network properties normalized? If so, state which ones *

No

Other:

1. How were the individual-specific networks used in the statistical modelling process? *

The full network was incorporated into the prognostic model(s)

Graph-theoretic attributes of the networks were included into a predictive model.

The individual-specific networks were only assessed for descriptive purposes but did not contribute to the prognostic model

The correlation coefficient between graph-theoretic attributes and the outcome is computed

Hypothesis testing was performed between two outcome groups.

The dynamic change of the network structure is assessed in multiple outcome groups.

Other:

1. Type of the outcome(s) *

Binary Continuous Time-to-event Ordinal Nominal

Rate

Other:

1. Outcome variables *
2. Additional information

**Advanced predictive modelling?**

1. Was a more complex approach taken regarding predictive modelling than hypothesis testing for group differences or the computation of a correlation coefficient?

Yes *(Continue with question 56)*

No *(Continue with question 76)*

Other:

*(Continue with question 56)*

This section focuses on question regarding the predictive model build based on the graph-theoretic attributes from the individual-specific networks

Predictive Model

1. Aim of the model *
2. Method(s) used for developing the prognostic model *

(e.g. linear/logistic regression, Cox regression, random forest, neural networks, support vector machines)

1. Can the method handle covariates (e.g. clinical information) that you want to adjust for? *

Yes No

Unclear

Other:

1. List the graph-theoretic attributes that were used as candidate predictors in the predictive model

In case of univariable analysis: leave the field empty

1. List additional attributes (e.g. clinical information, demographic features) that were used as candidate predictors in the prognostic model

In case of univariable analysis: leave the field empty

1. Does the selected methodology allow the quantification of the effect sizes of the graph-theoretical features?

Yes No

Unclear

Other:

1. Was any form of variable selection or reduction performed? *

Variable selection is not considered

Regression regularization techniques (e.g. Lasso, Ridge, Best subset)

Stepwise variable elimination procedure

Biological knowledge

Variable selection based on prior univariable analysis Principal component analysis (PCA)

Other:

1. Were global or local network features used in the model? *

i.e. global network features summarize structural aspects of the entire network, whereas local features mostly aim to capture spatial information of a node and its neighborhood

Global Local

Global and local Unclear

Other:

1. What was the final set of model predictors (graph-theoretic and non-graph- theoretic features)?

In case of univariable analysis: leave the field empty

1. Were specific network characteristics highlighted as particularly relevant predictors? If so, which ones?
2. Which other methods was the model compared to and did it show superior performance in terms of prediction accuracy? *

No other methods Other:

1. Additional information?

Validation?

1. Was the presented modelling approach validated?

Yes *(Continue with question 69)*

No *(Continue with question 76)*

Other:

Validation and Results

1. Type of validation performed for the model *

Internal External

Internal and external None

Other:

1. How was the model construction process validated? *
2. How was the discriminative performance of the model evaluated?
3. If predictions were made: which data was used for prediction? *

Apparent Internal External

No predictions Other:

1. If regression was performed: was the accuracy of the estimated regression coefficients considered?

e.g. MSE, bias, compared to literature, ...

Regression not used Unclear

No

Other:

1. Did any of network attributes show a statistical significant association with the outcome? If so, which ones?
2. Additional information

**Simulations?**

1. Was the performance of the prognostic model evaluated using simulated data? *

Yes

No *(Continue with question 86)*

**Simulation Study**

1. Were the simulations based on a real dataset? *

Yes No

Other:

1. How many simulation settings were considered? *
2. Which variables were varied for the different simulation settings? *
3. Describe the data-generating mechanism *

i.e. distribution of the outcome, data-generation of the network, distribution of additional individual-specific features (e.g. clinical information), correlation structure

1. How was the total performance of the model evaluated*?

e.g. C-index, R-squared, ...

1. Was a null situation considered? *

Yes No

Other:

1. Was a range of effect sizes considered? If yes, how was the strength of the effect quantified? *

No

Other:

1. Were scenarios considered in which the proposed method did not work? *
2. Were simulations performed to compare the method including the graph- theoretic framework with other methods? *

No

Other:

**Additional information**

1. State software, programming language and/or packages used *

Select software/programming languages used and if R was used, the mentioned R-packages.

Nothing stated R

Matlab Python SPSS

Brain Connectivity Toolbox Brainnetome Atlas

Automated Anatomical Labelling (AAL) Atlas

Other:

1. Is the data freely available? *

No Yes

Upon request

Link for download provided Anonymized data

Authors state yes but no further information

Other:

1. Is the code freely available? *

Yes No

Upon request

Other:

1. Were any limitations of the study stated? *

i.e. biological, technical or statistical nature? If statistical limitations state which ones.

1. Additional information?
2. Rank how relevant the study seems for the scoping review. *

Papers of high relevance will be prioritized post screening. Mildly relevant paper showcase a common approach that can be summarized.

1 2 3 4 5 6 7 8 9 10

Mildly relevant Highly relevant

**Literature**

1. Was any further interesting literature cited? If so, please provide the full citation(s)

This content was not created by Google and is not endorsed by Google.

[Formulare](https://www.google.com/forms/about/?utm_source=product&utm_medium=forms_logo&utm_campaign=forms)
